# Bigelovii A Protects against Lipopolysaccharide-Induced Acute Lung Injury by Blocking NF-*κ*B and CCAAT/Enhancer-Binding Protein *δ* Pathways

**DOI:** 10.1155/2016/9201604

**Published:** 2016-04-19

**Authors:** Chunguang Yan, Fuqin Guan, Yanfei Shen, Huifang Tang, Dong Yuan, Hongwei Gao, Xu Feng

**Affiliations:** ^1^Department of Pathogenic Biology and Immunology, Medical School of Southeast University, Nanjing 210009, China; ^2^Research Center for Natural Products Chemistry, Institute of Botany, Jiangsu Province and Chinese Academy of Sciences, Nanjing 210004, China; ^3^Department of Bioengineering, Medical School of Southeast University, Nanjing 210009, China; ^4^Zhejiang Respiratory Drugs Research Laboratory of the State Food and Drug Administration of China, School of Medicine, Zhejiang University, Hangzhou, Zhejiang 310058, China; ^5^Department of Intensive Care, Jintan Hospital, Jiangsu University, Changzhou, China; ^6^Center for Experimental Therapeutics and Reperfusion Injury, Department of Anesthesiology, Perioperative & Pain Medicine, Brigham and Women's Hospital, Harvard Medical School, Boston, MA 02115, USA

## Abstract

Optimal methods are applied to acute lung injury (ALI) and the acute respiratory distress syndrome (ARDS), but the mortality rate is still high. Accordingly, further studies dedicated to identify novel therapeutic approaches to ALI are urgently needed. Bigelovii A is a new natural product and may exhibit anti-inflammatory activity. Therefore, we sought to investigate its effect on lipopolysaccharide- (LPS-) induced ALI and the underlying mechanisms. We found that LPS-induced ALI was significantly alleviated by Bigelovii A treatment, characterized by reduction of proinflammatory mediator production, neutrophil infiltration, and lung permeability. Furthermore, Bigelovii A also downregulated LPS-stimulated inflammatory mediator expressions* in vitro*. Moreover, both NF-*κ*B and CCAAT/enhancer-binding protein *δ* (C/EBP*δ*) activation were obviously attenuated by Bigelovii A treatment. Additionally, phosphorylation of both p38 MAPK and ERK1/2 (upstream signals of C/EBP*δ* activation) in response to LPS challenge was also inhibited by Bigelovii A. Therefore, Bigelovii A could attenuate LPS-induced inflammation by suppression of NF-*κ*B, inflammatory mediators, and p38 MAPK/ERK1/2—C/EBP*δ*, inflammatory mediators signaling pathways, which provide a novel theoretical basis for the possible application of Bigelovii A in clinic.

## 1. Introduction

Acute lung injury (ALI) and the acute respiratory distress syndrome (ARDS) are important problems in human beings, leading to 75,000 deaths each year in the United States [1]. To dissect the molecular mechanisms of ALI and ARDS, a number of animal models have been developed, among which LPS-induced ALI is the most popular model [2]. In experimental ALI, alveolar macrophages are activated, leading to robust secretion of proinflammatory mediators, such as IL-6, MCP-1, and MIP-1*α*. These proinflammatory mediators contribute to the following transmigration of polymorphonuclear leukocytes (PMNs) from bloodstream into lung tissues. Then, activated PMNs produce reactive nitrogen and oxygen species and proteases, which injure pulmonary parenchyma. The end results are increased lung microvascular permeability, intrapulmonary hemorrhage, and accumulation of protein rich edema fluid and fibrin [3, 4]. Moreover, all these features can be observed in patients suffering from ARDS [5, 6]. Currently, there are no clinical therapeutic agents that could be used to protect pulmonary tissues from injury or promote tissue repair. Therefore, studies should be conducted to identify novel methods that could inhibit ALI or accelerate resolution of ALI. One of the strategies is to suppress generation of proinflammatory mediators, which are the initiators of ALI.

C/EBP*δ* is an important transcription factor that regulates a variety of gene expressions critical to various inflammation-associated diseases [7–9], including LPS-induced acute lung injury [10, 11]. During LPS stimulation, C/EBP*δ* expression is elevated, resulting in its increased accumulation in nucleus, which is indispensable for full expressions of inflammation-related genes, such as IL-6 and MIP-2 [10, 11]. Moreover, it has been demonstrated that mitogen-activated protein kinases (MAPKs), including p38 MAPK and ERK1/2, play essential roles in inflammatory responses [12, 13], which are upstream factors of C/EBP*δ* activation [11]. In addition, NF-*κ*B, as a nuclear transcription factor, also participates in LPS-induced generation of proinflammatory mediators [14–16]. When bound by the I*κ*B family proteins, NF-*κ*B is inactivated and sequestered in cytoplasmic compartment. Once certain inflammatory stimulus appears, degradation of I*κ*B family proteins is induced, which leads to the release of NF-*κ*B, followed by its translocation from cytosol to nucleus, where it initiates transcription of proinflammatory genes. Previous studies have validated the involvement of NF-*κ*B in LPS-induced ALI [16]. Therefore, downregulation of C/EBP*δ* and/or NF-*κ*B activation might be a promising strategy for therapy of ALI.

Our previous studies have shown the chemical structure of a new nor-Oleanane type triterpene saponin (Bigelovii A) isolated from the dried herbs of* Salicornia bigelovii* Torr. [17], and its antitumor activity has been observed in HL-60, MCF-7, and HepG2 cells [18]. However, the anti-inflammatory activity of Bigelovii A has not been examined. Because the core structure of Bigelovii A is triterpenoid that has inhibitory activity on inflammation [19, 20], in the current study, we sought to determine the effect of Bigelovii A on acute pulmonary inflammation stimulated by LPS. Our data suggested that Bigelovii A treatment reduced proinflammatory mediator expressions in bronchoalveolar lavage fluid (BALF), accompanied with decreased accumulation of PMNs in lungs. For mechanistic investigation, MH-S cells were applied. We found that LPS-mediated inflammatory cytokine and chemokine generation could also be suppressed by Bigelovii A in the cells, which was related to decreased C/EBP*δ* and NF-*κ*B transcriptional activities. Furthermore, we provided the evidence that inhibition of C/EBP*δ* activation by Bigelovii A was associated with downregulation of p38 MAPK and ERK1/2 phosphorylation.

## 2. Materials and Methods

### 2.1. Cells, Animals, and Reagents

Pathogen-free C57BL/6 mice were obtained from Model Animal Research Center of Nanjing University (Nanjing, China) and maintained in a specific pathogen-free facility. All animal studies were performed in accordance with guidelines approved by the Institutional Animal Care and Use Committee of Southeast University. MH-S cells, derived from mouse alveolar macrophages, were purchased from American Type Culture Collection (Manassas, VA, USA) and maintained in RPMI 1640 medium supplied with 10% fetal bovine serum (FBS) and 0.01 M HEPES. THP-1 cells (ATCC), which are human-derived monocytes, were cultured in RPMI 1640 medium with 10% FBS. ELISA kits for mouse IL-6, MCP-1, MIP-1*α*, and MIP-2 were all obtained from R&D Systems (Minneapolis, MN, USA). As described previously, Bigelovii A was extracted from the dried herbs of* Salicornia bigelovii* Torr. [17].

### 2.2. LPS-Stimulated Acute Lung Injury

Mice were firstly anesthetized with intraperitoneal administration of 1.5% sodium pentobarbital. The animals were then challenged by intratracheal instillation of 50 *μ*g LPS dissolved in PBS [2, 10, 11]. Control mice received airway administration of PBS. 18 h or 60 h after LPS stimulation, BAL fluids were obtained, and ELISA was performed by using cell-free supernatants. When employed, Bigelovii A (10 mg/kg) was intraperitoneally instilled 60 min before LPS treatment.

### 2.3. Analysis of Myeloperoxidase (MPO) Content

Pulmonary tissues were flushed* via* the right ventricle with 1 mL of PBS. For MPO content assay, lungs were homogenized in lysis buffer containing 0.5% hexadecyltrimethylammonium bromide, 50 mM potassium phosphate buffer, and 5 mM EDTA. Then, lung samples were sonicated, and cell-free supernatants were collected. 10 *μ*L of the supernatants were incubated with the MPO assay buffer containing 5 *μ*g/mL H_2_O_2_, 167 *μ*g/mL* o*-dianisidine dihydrochloride, and 100 mM potassium. MPO level was evaluated by measurement of the optical density change at 450 nm over 3 min utilizing a 96-well plate reader.

### 2.4. BAL Fluid Analysis

18 or 60 hours after initiation of LPS-induced acute pulmonary injury, mice were exsanguinated, and the thorax was opened. 1 mL of ice-cold PBS was injected* via* an intratracheal cannula. The recovered bronchoalveolar lavage fluid was centrifuged at 3000 rpm for 5 min, and cell pellets were resuspended in 1 mL of HBSS containing 0.5% BSA. Differential cell assays were conducted by Diff-Quik-stained cytospin preparations (Dade, Düdingen, Switzerland) counting a total of 300 cells per slide in randomly selected high-powered fields (×1000). Cell-free supernatants were used for measuring proinflammatory mediator production and the albumin level (an indicator of microvascular permeability).

### 2.5. Histological Examination

18 h after pulmonary deposition of LPS, the lungs were fixed by intratracheal injection of 1 mL 10% neutral phosphate-buffered formalin. The pulmonary tissues were then removed and maintained in 10% buffered formalin solution for histological analysis by tissue sectioning and staining with haematoxylin and eosin (H&E).

### 2.6. RNA Extraction and Real Time PCR

RNA was isolated by using Trizol purchased from Invitrogen. We then conducted reverse transcription to obtain cDNAs. Real time PCR was performed by using the following primers: mouse IL-6, 5′ primer, 5′-AGT TGC CTT CTT GGG ACT GA-3′ and 3′ primer, 5′-TCC ACG ATT TCC CAG AGA AC-3′; mouse MCP-1, 5′ primer, 5′-AGG TCC CTG TCA TGC TTC TG-3′ and 3′ primer, 5′-TCT GGA CCC ATT CCT TCT TG-3′; mouse MIP-1*α*, 5′ primer, 5′-ATG AAG GTC TCC ACC ACT GC-3′ and 3′ primer, 5′-CCC AGG TCT CTT TGG AGT CA-3′; mouse MIP-2, 5′ primer, 5′-AGT GAA CTG CGC TGT CAA TG-3′ and 3′ primer, 5′-TTC AGG GTC AAG GCA AAC TT-3′; human IL-6, 5′ primer, 5′-AGT CCT GAT CCA GTT CCT GC-3′ and 3′ primer, 5′-AAG CTG CGC AGA ATG AGA TG-3′; human IL-8, 5′ primer, 5′-CAG TTT TGC CAA GGA GTG CT-3′ and 3′ primer, 5′-ACT TCT CCA CAA CCC TCT GC-3′.

### 2.7. Plasmids and Luciferase Analysis

*κ*B-Luc—luciferase expression driven by NF-*κ*B (cat. number: D2206)—was purchased from Beyotime Biotechnology, China. pRL-TK vector (cat. number: E2231) was from Promega Corporation, which was used as an internal control reporter in combination with any experimental reporter vector to cotransfect MH-S cells. The DEI_4_-Luc harboring 4 copies of a C/EBP binding site and MCP-1-Luc—luciferase expression driven by MCP-1 promoter—were kindly provided by Dr. Richard C. Schwartz (Michigan State University). Total of 0.5 *μ*g plasmids including 0.45 *μ*g reporter vector and 0.05 *μ*g pRL-TK were introduced into MH-S cells by using Fugene® 6 Transfection Reagent as recommended by the manufacturer. Forty-eight hours later, cells were treated with or without 100 ng/mL of LPS for 4 hours. The cells were then lysed and luciferase expressions were measured by using Dual-Luciferase Reporter Assay System (Promega, Madison, WI, USA).

### 2.8. Western Blot

Total proteins were extracted from MH-S cells by using RIPA buffer, and 60 *μ*g proteins were separated by SDS-PAGE, transferred, and immobilized on a PVDF membrane. The membrane was then blocked by using 5% nonfat milk solution. Rabbit anti-NF-*κ*B p-p65 antibody (cat. number: 3033S), rabbit anti-p-p38 antibody (cat. number: 9211S), rabbit anti-p-p44/42 antibody (cat. number: 9101S), rabbit anti-p38 antibody (cat. number: 9212S), rabbit anti-p44/42 antibody (cat. number: 9102S), rabbit anti-C/EBP*δ* antibody (cat. number: 2318), and rabbit anti-GAPDH antibody (cat. number: 2118) were used. All of these antibodies were obtained from Cell Signaling Technology and diluted 1000-fold with 5% nonfat milk solution. Identification of the target proteins was conducted by utilizing the Enhanced Chemiluminescence kit (Amersham Biosciences UK, Buckinghamshire, UK).

### 2.9. Statistical Assay

All values were expressed as the mean ± SEM. Significance was assigned where *p* < 0.05. Data sets were analyzed using Student's *t*-test or one-way ANOVA, with individual group means being compared with the Student-Newman-Keuls multiple comparison test.

## 3. Results

### 3.1. LPS-Induced Acute Lung Injury Was Alleviated by Bigelovii A Treatment

**Figure 1:**
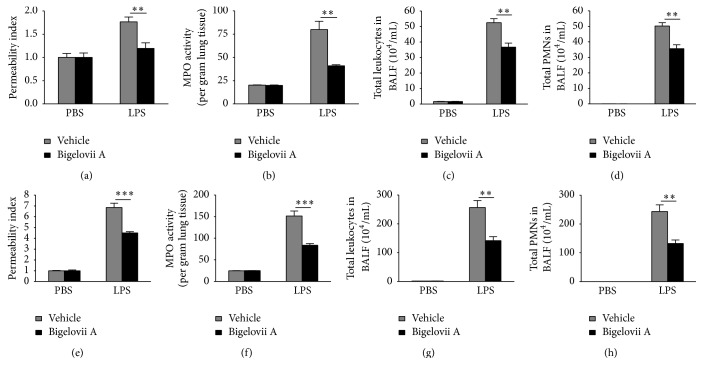
Effect of Bigelovii A on acute lung injury (ALI) induced by airway administration of LPS. ALI was induced by intratracheal administration of LPS. 18 h (a–d) or 60 h (e–h) later, BAL fluids were extracted, and the whole lungs were harvested. (a and e) ELISA was conducted to detect mouse albumin levels in BAL fluids, and the permeability index was expressed as the ratio of the albumin in the LPS-treated lungs versus that in the PBS-treated lungs of same type of mice. (b and f) MPO contents in whole lung tissues were determined. Total cell (c and g) and neutrophil (d and h) accumulation in BAL fluids were estimated. Results are reflected by means ± SEM [*n* = 3 for Vehicle plus PBS group (negative control) or Bigelovii A+PBS group, *n* = 6 for LPS+Vehicle group (positive control), and *n* = 5 for LPS+Bigelovii A group]. *∗∗* and *∗∗∗* indicated statistically significant difference, *p* < 0.01 and 0.001, respectively.

The core structure of Bigelovii A is triterpenoid that has inhibitory activity on inflammation [19, 20]. In the current study, we sought to determine the effect of Bigelovii A on acute pulmonary injury stimulated by LPS. As shown in Figures 1(a) and 1(e), the microvascular permeability index of mice receiving airway administration of LPS was obviously augmented compared with negative control. However, the i.p. injection of Bigelovii A (10 mg/kg) led to significant reduction in lung permeability index (Figures 1(a) and 1(e)).

LPS-induced acute lung injury is a neutrophil-dependent process, so neutrophil infiltration reflects to what extent the pulmonary tissues are damaged. We evaluated the effect of Bigelovii A on neutrophil accumulation and found that the natural product treatment resulted in great decrease in lung MPO content (an indicator of neutrophil counts) when compared with the positive control (Figures 1(b) and 1(f)). We further observed that mice treated by Bigelovii A and LPS together exhibited significant alleviation of total contents of white blood cells and PMNs obtained from BAL fluids compared to mice challenged by LPS alone (Figures 1(c), 1(d), 1(g), and 1(h)).

**Figure 2:**
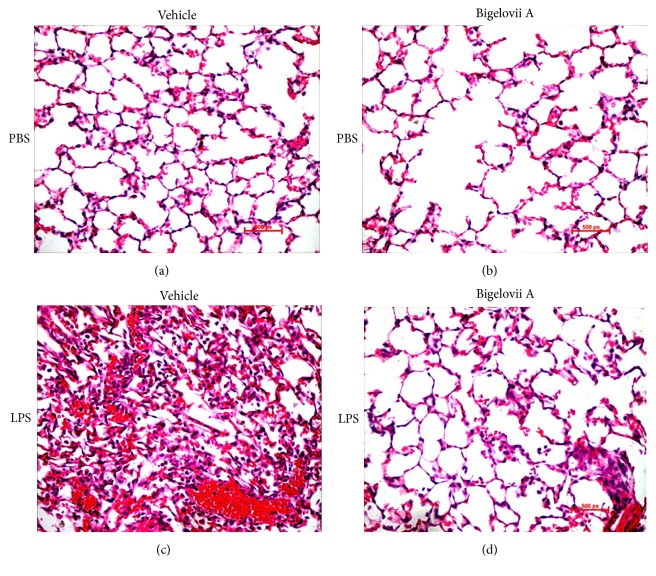
Effect of Bigelovii A on LPS-induced histological change. ALI was induced by intratracheal administration of LPS. 18 h later, lung tissues were prepared as described in Section 2 (original magnification ×400). Lung sections were Vehicle plus PBS (negative control) (a), Bigelovii A+PBS (b), LPS+Vehicle (positive control) (c), and LPS+Bigelovii A (d), respectively.

To further estimate whether LPS-induced acute lung injury could be relieved by the natural product, histological assays were conducted. As shown in Figure 2(b), mice treated by Bigelovii A alone displayed normal pulmonary architecture without any signs of inflammatory responses. LPS stimulation led to pulmonary hemorrhage, edema, and accumulation of inflammatory cells, especially neutrophils (Figure 2(c)). However, mice receiving Bigelovii A treatment showed obvious attenuation of the injury-related characteristics 18 h after airway deposition of LPS (Figure 2(d)).

**Figure 3:**
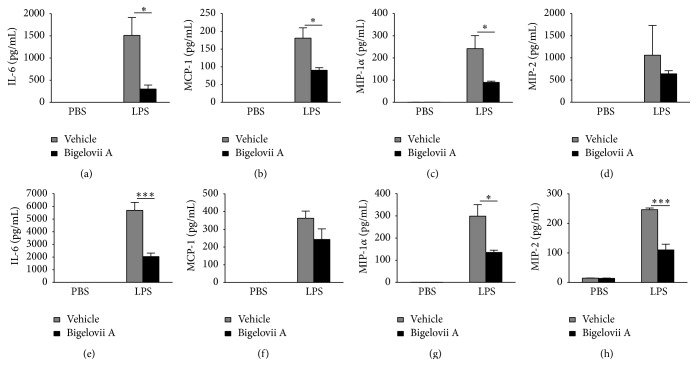
Effect of Bigelovii A on LPS-induced production of inflammatory mediators in BAL fluids. ALI was induced by intratracheal administration of LPS. 18 h or 60 h later, BAL fluids were extracted and generation of IL-6 (a and e), MCP-1 (b and f), MIP-1*α* (c and g), and MIP-2 (d and h) was measured. Results are reflected by means ± SEM [*n* = 3 for Vehicle plus PBS group (negative control) or Bigelovii A+PBS group, *n* = 6 for LPS+Vehicle group (positive control), and *n* = 5 for LPS+Bigelovii A group]. *∗* and *∗∗∗* indicated statistically significant difference, *p* < 0.05 and 0.001, respectively.

Production of various proinflammatory mediators is a prerequisite for LPS-induced acute lung injury, so we determined several cytokine and chemokine production in BAL fluids. As expected, almost no production of proinflammatory mediators was detected in BAL fluids harvested from mice that were not challenged by LPS (Figures 3(a)–3(h)). However, mice undergoing airway deposition of LPS exhibited amplified generation of IL-6, MCP-1, MIP-1*α*, and MIP-2 compared with the negative control (Figures 3(a)–3(h)). The contents of all these proinflammatory mediators were obviously reduced in mice treated by Bigelovii A (Figures 3(a)–3(h)). These data correlated with the above depicted features—reduced microvascular permeability, neutrophil influx, and histological alteration.

### 3.2. Bigelovii A Decreased Inflammation-Associated Gene Expressions in LPS-Treated MH-S Cells

**Figure 4:**
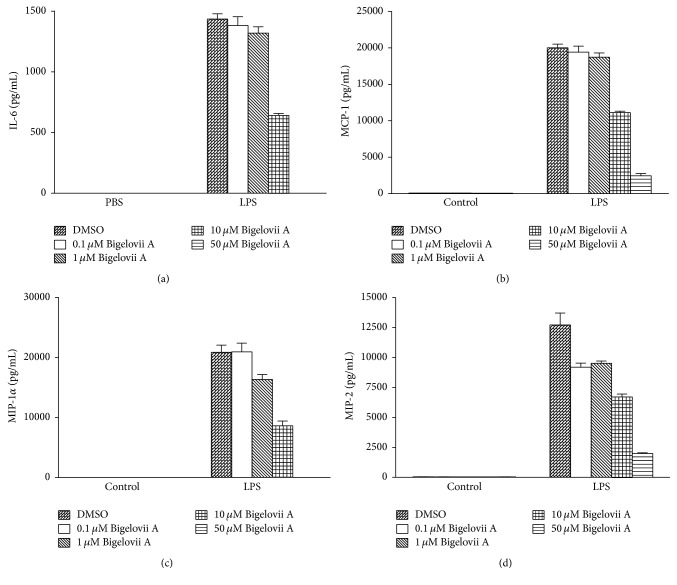
Effect of Bigelovii A on cytokine and chemokine secretion during LPS-induced inflammation in MH-S cells. MH-S cells were pretreated with the indicated dose of Bigelovii A for 1 h, followed by treatment with PBS or 100 ng/mL of LPS for 6 h. Then cell-free supernatants were harvested, and ELISA was conducted to measure production of IL-6 (a), MCP-1 (b), MIP-1*α* (c), and MIP-2 (d). Results are reflected by means ± SEM (*n* = 6).

**Figure 5:**
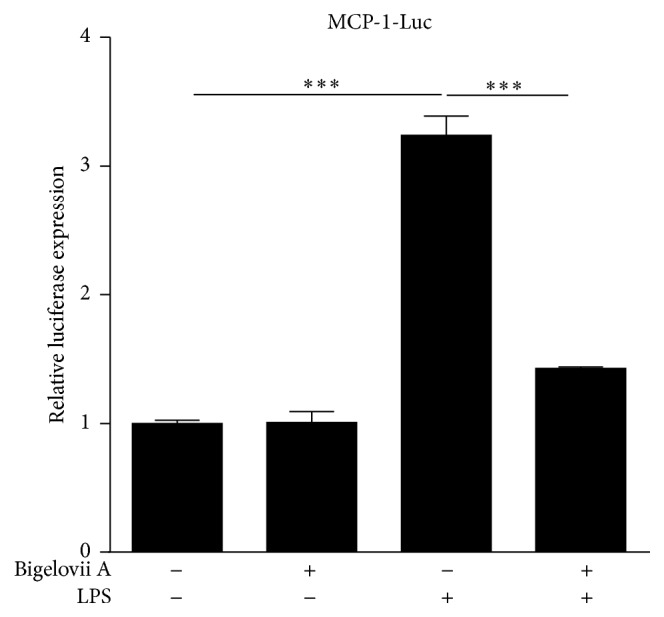
Effect of Bigelovii A on MCP-1 promoter-driven luciferase expression during LPS-induced inflammation in MH-S cells. MH-S cells were transiently transfected with total of 0.5 *μ*g indicated plasmids. 48 h after transfection, the cells were pretreated with or without 10 *μ*M of Bigelovii A for 1 h, followed by treatment with PBS or 100 ng/mL of LPS. 4 h later, the cells were lysed, and luciferase activity was measured. Luminometer values were normalized for expression from a cotransfected pRL-TK gene and negative control sequentially. Results are reflected by means ± SEM (*n* = 3). *∗∗∗* indicated statistically significant difference—*p* < 0.001. Negative control: cells treated by Vehicle plus PBS. Positive control: cells receiving LPS plus Vehicle treatment.

**Figure 6:**
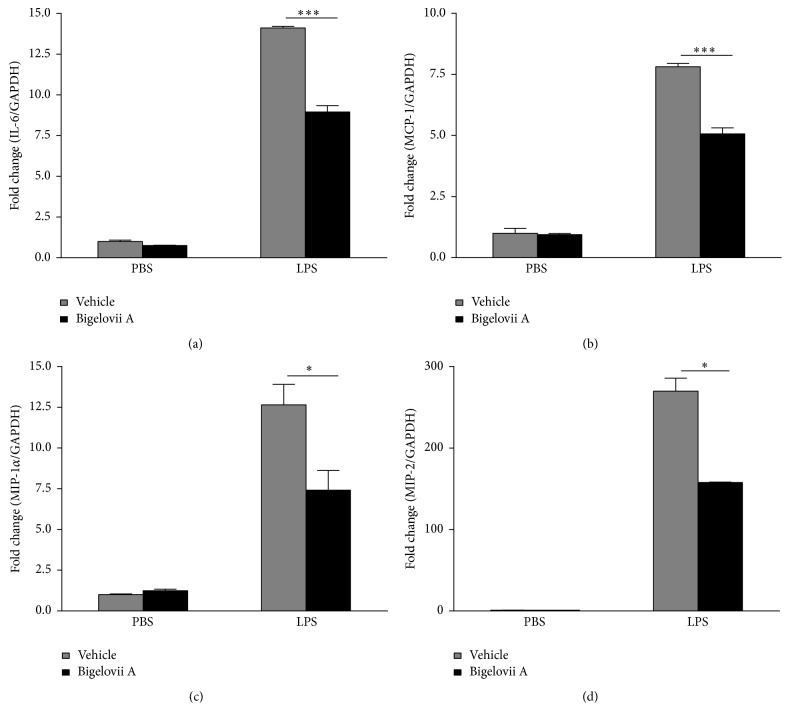
Effect of Bigelovii A on cytokine and chemokine transcription during LPS-induced inflammation in MH-S cells. MH-S cells were pretreated with 10 *μ*M of Bigelovii A for 1 h, followed by treatment with PBS or 100 ng/mL of LPS for 3 h. mRNAs were then extracted. Real time PCR was conducted to measure the mRNA levels of IL-6 (a), MCP-1 (b), MIP-1*α* (c), and MIP-2 (d). Results are reflected by means ± SEM (*n* = 3). *∗* and *∗∗∗* indicated statistically significant difference, *p* < 0.05 and 0.001, respectively. Negative control: cells treated by Vehicle plus PBS. Positive control: cells receiving LPS plus Vehicle treatment.

**Figure 7:**
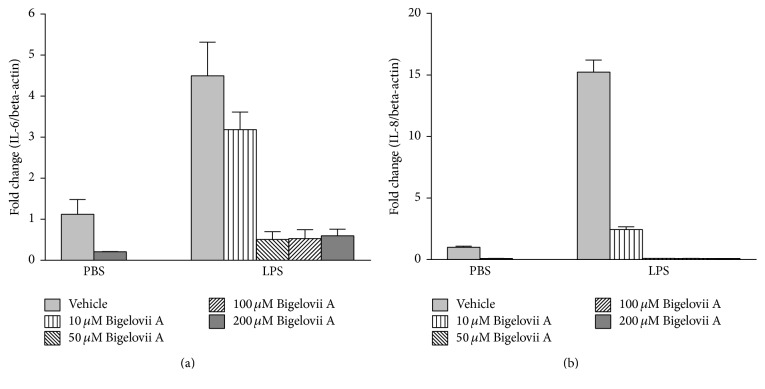
Effect of Bigelovii A on cytokine and chemokine expressions during LPS-induced inflammation in human-derived monocytes. THP-1 cells were pretreated with the indicated dose of Bigelovii A for 1 h, followed by treatment with PBS or 100 ng/mL of LPS for 2 h. mRNAs were then extracted. Real time PCR was conducted to measure the mRNA levels of IL-6 (a) and IL-8 (b). Results are reflected by means ± SEM (*n* = 3).

Alveolar macrophages are critical initiators of acute lung inflammatory responses [21–25]. Our recent studies also find that alveolar macrophage activation is involved in LPS-stimulated acute lung inflammation [11]. Therefore, MH-S cells were used in our present study to evaluate the influence of Bigelovii A on inflammatory responses* in vitro* and the mechanisms whereby the natural product could suppress inflammation. As shown in Figures 4(a)–4(d), secretion of IL-6, MCP-1, MIP-1*α*, and MIP-2 was dramatically stimulated by LPS in MH-S cells. However, Bigelovii A obviously reduced LPS-induced production of the above proinflammatory mediators in a dose-dependent manner (Figures 4(a)–4(d)). We found that 10 *μ*M of Bigelovii A was the minimal dose that should be used to significantly inhibit LPS-induced inflammation in MH-S cells. Thus, in the following experiments related to MH-S cells, 10 *μ*M of Bigelovii A was applied. By conducting luciferase assay, we observed that LPS stimulation increased MCP-1 promoter-driven luciferase activity over 4-fold compared with the negative control group (Figure 5). However, Bigelovii A treatment considerably inhibited LPS-triggered luciferase expression (~56%) in MH-S cells (Figure 5), which indicated that Bigelovii A might downregulate transcription of inflammatory genes. We next examined whether Bigelovii A treatment played a negative role in inflammation-related gene expression in LPS-activated MH-S cells at mRNA level. The data showed that transcription of the proinflammatory mediators was dramatically stimulated by LPS in the cells (Figures 6(a)–6(d)). However, compared with positive control groups, Bigelovii A significantly reduced LPS-induced production of IL-6 (~36%), MCP-1 (~36%), MIP-1*α* (~50%), and MIP-2 (~30%) (Figures 6(a)–6(d)). Moreover, we tested whether the phenomena observed in murine cells were applicable to human-derived cells and found that IL-6 and IL-8 expressions were also dose-dependently inhibited by Bigelovii A in LPS-treated THP-1 cells (Figures 7(a) and 7(b)).

### 3.3. Retardation of NF-*κ*B Transcriptional Activity Was Induced by Bigelovii A in LPS-Treated MH-S Cells

**Figure 8:**
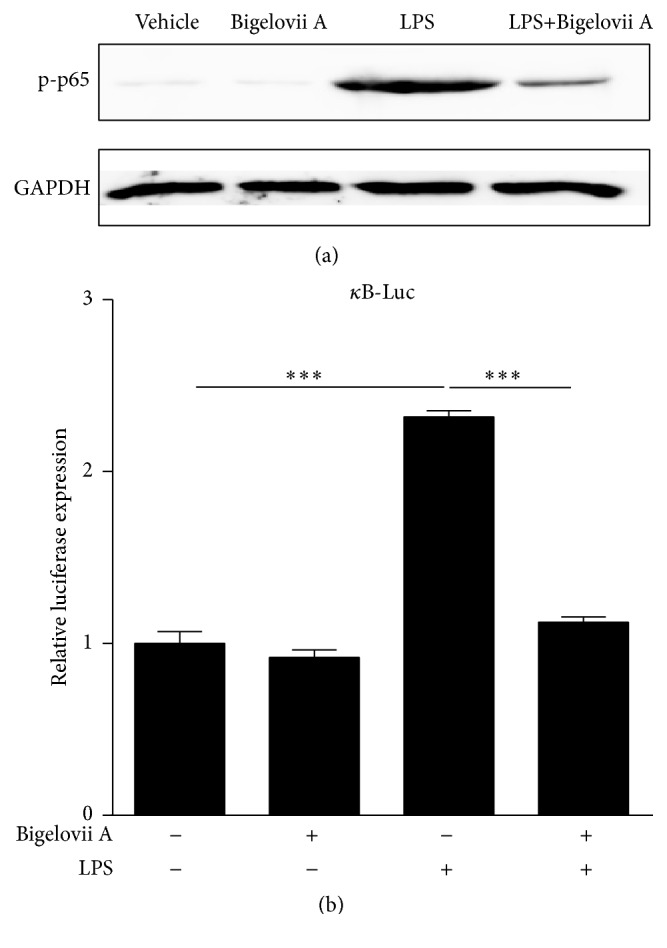
Effect of Bigelovii A on NF-*κ*B activation during LPS-induced inflammation in MH-S cells. (a) MH-S cells were pretreated with or without 10 *μ*M of Bigelovii A for 1 h, followed by treatment with PBS or 100 ng/mL of LPS for 3 h, and total proteins were isolated. Western blot was performed by using antibodies against NF-*κ*B p-p65 and GAPDH, respectively. (b) MH-S cells were transiently transfected with total of 0.5 *μ*g indicated plasmids. 48 h after transfection, the cells were pretreated with or without 10 *μ*M of Bigelovii A for 1 h, followed by treatment with PBS or 100 ng/mL of LPS. 4 h later, the cells were lysed, and luciferase activity was measured. Luminometer values were normalized for expression from a cotransfected pRL-TK gene and negative control sequentially. Results are reflected by means ± SEM (*n* = 3). *∗∗∗* indicated statistically significant difference, *p* < 0.001. Negative control: cells treated by Vehicle plus PBS. Positive control: cells receiving LPS plus Vehicle treatment.

The above data demonstrated the downregulation of inflammation-associated gene expression by Bigelovii A at transcription level (Figures 6(a)–6(d)). As a pivotal transcription factor, NF-*κ*B is involved in a variety of inflammatory gene transcriptions. Therefore, we sought to estimate the influence of Bigelovii A on LPS-triggered NF-*κ*B activation in MH-S cells. To that end, we first performed Western blot analysis to detect whether NF-*κ*B p65 phosphorylation was blocked by Bigelovii A. As shown in Figure 8(a), the basal level of phosphorylated NF-*κ*B p65 was not affected by Bigelovii A treatment in MH-S cells (lanes 1 and 2), and LPS treatment obviously amplified phosphorylation of p65 (lanes 1 and 3). When Bigelovii A was used, LPS-induced elevation of p65 phosphorylation was greatly alleviated (Figure 8(a), lanes 3 and 4). We then evaluated whether NF-*κ*B transcriptional activity was influenced by Bigelovii A in the cells treated with LPS. As shown in Figure 8(b), LPS treatment induced luciferase expression by over 2.3-fold. However, Bigelovii A treatment led to an over 52% reduction in LPS stimulation of luciferase expression (Figure 8(b)), which suggested that LPS-stimulated NF-*κ*B activation was negatively regulated by the natural product.

### 3.4. The LPS-Activated Signaling Pathway (p38 MAPK/ERK—C/EBP*δ*: Proinflammatory Mediators) Was Disrupted by Bigelovii A in MH-S Cells

**Figure 9:**
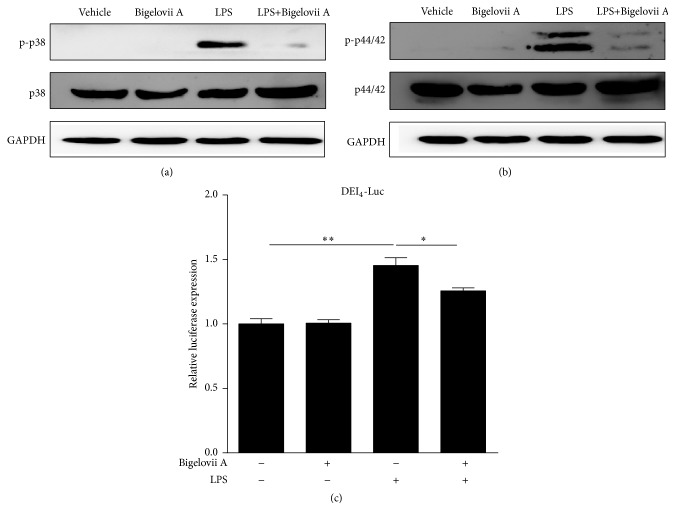
Effect of Bigelovii A on p38 MAPK/ERK1/2—C/EBP*δ* signaling pathway during LPS-induced inflammation in MH-S cells. (a) and (b) MH-S cells were pretreated with or without 10 *μ*M of Bigelovii A for 1 h, followed by treatment with PBS or 100 ng/mL of LPS for 15 min, and total proteins were isolated. Western blot was performed by using antibodies against p-p38, p38, p-p44/42, p44/42, and GAPDH, respectively. (c) MH-S cells were transiently transfected with total of 0.5 *μ*g indicated plasmids. 48 h after transfection, the cells were pretreated with or without 10 *μ*M of Bigelovii A for 1 h, followed by treatment with PBS or 100 ng/mL of LPS. 4 h later, the cells were lysed, and luciferase activity was measured. Luminometer values were normalized for expression from a cotransfected pRL-TK gene and negative control sequentially. Results are reflected by means ± SEM (*n* = 3). *∗* and *∗∗* indicated statistically significant difference, *p* < 0.05 and 0.01, respectively. Negative control: cells treated by Vehicle plus PBS. Positive control: cells receiving LPS plus Vehicle treatment.

Our previous studies have demonstrated the existence of the signaling pathway—p38 MAPK/ERK—C/EBP*δ*—proinflammatory mediators in LPS-treated alveolar macrophages [11]. To further identify the underlying mechanism whereby LPS-induced generation of proinflammatory mediators was disturbed by Bigelovii A, we examined the involvement of Bigelovii A in interference with the mentioned signaling pathway. As shown in Figures 9(a) and 9(b), the basal level of both p-p38 MAPK and p-p44/42 almost could not be detected (lanes 1 and 2). LPS stimulation considerably elevated the contents of both phosphorylated MAPK members (Figures 9(a) and 9(b), lanes 1 and 3). However, Bigelovii A treatment led to decreases in LPS-induced accumulation of p-p38 MAPK and p-p44/42 in MH-S cells (Figures 9(a) and 9(b), lanes 3 and 4), which indicated that C/EBP*δ* activation might be downregulated by Bigelovii A. We next estimated the influence of Bigelovii A on LPS induction of C/EBP transcription activity, and luciferase assay was performed. We found that C/EBP-dependent (DEI_4_-Luc) luciferase activity was greatly induced by LPS; however, Bigelovii A treatment considerably reduced LPS stimulation of luciferase expression (Figure 9(c)).

### 3.5. NF-*κ*B p-p65 and C/EBP*δ* Levels Were Impaired by Bigelovii A Treatment after Intratracheal Injection of LPS

**Figure 10:**
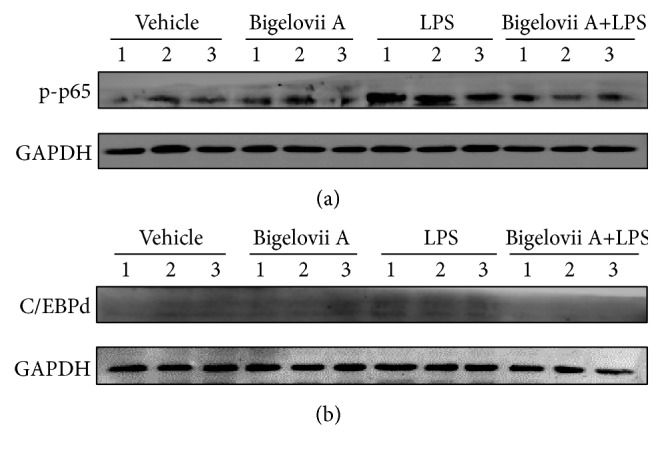
Effect of Bigelovii A on NF-*κ*B activation and C/EBP*δ* expression during LPS-induced acute lung injury. Lung injury was induced by intratracheal administration of LPS. 3 h later, total proteins were extracted from whole lung tissues, and Western blot were performed by using antibodies against NF-*κ*B p-p65, C/EBP*δ*, and GAPDH, respectively. Mice receiving Vehicle and PBS treatment were considered as negative controls, and mice treated by Vehicle plus LPS were used as positive control.

The above data have demonstrated the involvement of both NF-*κ*B p-p65 and C/EBP*δ* in Bigelovii A-mediated suppression of LPS-activated inflammation* in vitro*. We next tested the role of Bigelovii A in NF-*κ*B p-p65 and C/EBP*δ* accumulation in LPS-treated lungs by Western blot assays. As shown in Figures 10(a) and 10(b), NF-*κ*B p65 phosphorylation and C/EBP*δ* expression were greatly induced in lungs receiving LPS treatment when compared with the negative control. However, LPS-induced accumulation of both transcription factors was obviously blocked by Bigelovii A (Figures 10(a) and 10(b)), which was consistent with the data obtained from MH-S cells. Together, these data proved that Bigelovii A attenuated LPS-stimulated acute lung inflammatory responses through downregulation of NF-*κ*B p-p65 and C/EBP*δ* levels.

## 4. Discussion

Acute lung injury (ALI) and the acute respiratory distress syndrome (ARDS) are life-threatening disorders characterized by pulmonary edema and infiltration of inflammatory cells, especially neutrophils [26]. During ALI, robust expressions of a broad spectrum of cytokines and chemokines are induced, resulting in uncontrolled inflammation, which is considered as the key factor contributing to pulmonary injury [27]. Though optimal treatment methods are applied, the mortality caused by ALI varies between 35% and 60%, which is due to the lack of clinical therapeutic agents available to protect lung tissues from severe inflammation-mediated damage or accelerate resolution of pulmonary inflammatory reactivities [26]. Accordingly, further studies dedicated to identify novel therapeutic approaches to ALI are urgently needed [28–30].

Currently, natural products and their derivatives provide new views about treatment of inflammation-associated diseases, including acute lung injury [31–35]. We recently extracted Bigelovii A—a new nor-Oleanane type triterpene saponin—from the dried herbs of* Salicornia bigelovii* Torr. [17]. Its core structure is triterpenoid that exerts suppressive effect on inflammatory mediator expression [19, 20]. Thus, we examined the role of Bigelovii A in LPS-induced acute lung injury.

LPS-induced ALI in rodents is extensively used for mechanistic investigation of ALI and ARDS. In the model, intratracheal challenge of LPS leads to rapid activation of inflammatory cells, especially alveolar macrophages, resulting in robust formation of inflammatory mediators, and the subsequent neutrophil recruitment and tissue damage [2, 36]. By applying this model, we validated for the first time that LPS-induced acute lung inflammation and the following ALI were significantly inhibited by Bigelovii treatment. These were evidenced by decreased inflammatory mediator levels, neutrophil infiltration, and pulmonary microvascular leakage (permeability index) (Figures 1–3). Taken together, these data indicated that Bigelovii A negatively regulated LPS-induced acute inflammatory reactivities and damage in lung tissues.

Alveolar macrophages are critical initiators of acute lung inflammatory responses [21–25]. Using liposome encapsulated dichloromethylene diphosphonate, which can lead to alveolar macrophage depletion, Lentsch et al. demonstrate the fundamental role of macrophages in IgG immune complex-induced pulmonary damage [37]. Our recent studies also find that alveolar macrophage activation is involved in LPS-stimulated acute lung inflammation [11]. Therefore, MH-S cells were used in our present study to elucidate the influence of Bigelovii A on inflammatory responses* in vitro* and the mechanisms whereby the natural product could suppress inflammation. Our findings showed that Bigelovii A treatment obviously inhibited LPS-induced generation of proinflammatory mediators, including IL-6, MCP-1, MIP-1*α*, and MIP-2, at both mRNA and protein levels (Figures 4–6). Furthermore, we found that the transcriptional activities of both NF-*κ*B and C/EBP*δ* were significantly downregulated by Bigelovii A (Figures 8(b) and 9(c)). Both transcription factors are involved in a broad spectrum of inflammation-associated gene expressions, such as chemoattractants, cytokines, and their receptors. Previous studies have demonstrated the essential roles of NF-*κ*B and C/EBP*δ* in inflammatory responses, including acute pulmonary inflammation [9, 11, 16, 38]. Thus, reduced activation of both transcription factors might be the underlying mechanisms whereby Bigelovii A suppressed inflammatory mediator production and ensuing tissue injury in LPS-challenged lungs. Intriguingly, recent studies showed that the natural product, resolvin D1, could control resolution of acute inflammation in macrophages by regulating expression of specific microRNA targeting NF-*κ*B signaling [39, 40]. Whether the potential mechanism is also applied to regulation of NF-*κ*B and C/EBP*δ* signaling pathways by Bigelovii A remains a challenging question. Our data also provided evidence that Bigelovii A treatment resulted in obvious reduction of phosphorylated forms of both p38 MAPK and ERK1/2 in MH-S cells (Figures 9(a) and 9(b)). Influence of p38 MAPK and ERK1/2 on inflammation has been widely investigated, and our recent studies suggest that p38 MAPK and ERK1/2 positively regulate inflammatory responses by controlling C/EBP*δ* accumulation in nucleus [11]. Interestingly, our previous studies demonstrate that SOCS3 downregulates LPS-induced inflammatory gene expressions* via* reduction of C/EBP*δ* activation [10]. In the future, it is intriguing to conduct experiments to find out whether SOCS3—C/EBP*δ* signal, as an essential circuit, could be regulated by Bigelovii A during LPS-stimulated inflammation.

In summary, these data indicated that, during LPS-induced inflammatory responses, upstream signaling pathways, such as p38 MAPK and ERK1/2, were provoked, leading to activation of downstream transcription factors, including NF-*κ*B and C/EBP*δ*, which played central roles in expressions of proinflammatory mediators, and Bigelovii A could attenuate LPS-induced inflammation by suppression of NF-*κ*B, inflammatory mediators, and p38 MAPK/ERK1/2—C/EBP*δ*, inflammatory mediators signaling pathways, which provide a novel theoretical basis for the possible application of Bigelovii A in clinic.

## 5. Conclusions

**Figure 11:**
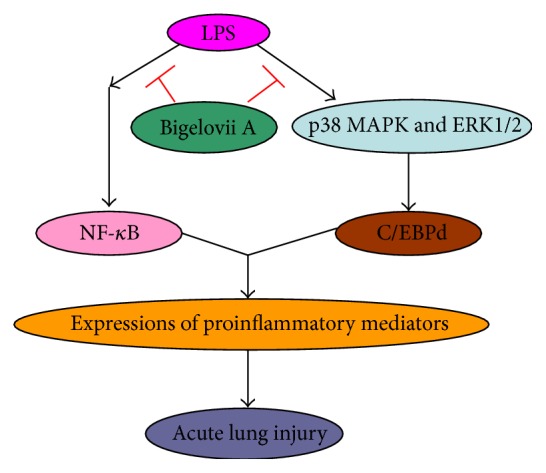
During LPS stimulation, both NF-*κ*B and p38 MAPK/ERK1/2—C/EBP*δ* pathways are activated, which contribute to production of proinflammatory mediators and acute lung injury. However, Bigelovii A, as a new discovered natural product from the dried herbs of* Salicornia bigelovii* Torr., can disrupt LPS induction of both signaling pathways, leading to attenuation of inflammatory responses.

Bigelovii A mitigated LPS-induced ALI by suppressing NF-*κ*B and CCAAT/enhancer-binding protein *δ* pathways (Figure 11).
